# Overweight & obese Australian adults and micronutrient deficiency

**DOI:** 10.1186/s40795-020-00336-9

**Published:** 2020-05-01

**Authors:** Jenny McKay, Suleen Ho, Monica Jane, Sebely Pal

**Affiliations:** School of Public Health, Faculty of Health Sciences, Curtin University, Perth, Western Australia

**Keywords:** Obesity, Micronutrients, Nutrient reference values (NRVs), Absorption, Metabolism, Bioavailability, Body mass index (BMI), Vitamins, Minerals, Deficiency

## Abstract

**Background:**

Micronutrients have been implicated as an important factor in regulating various metabolic processes and thus playing a role in the aetiology of obesity. Many studies have been conducted worldwide that clearly show a direct link between obesity and micronutrient deficiencies. The aim of this study was to assess the nutritional status of overweight and obese Australian adults to see if there were any associations between BMI and serum micronutrient levels.

**Methods:**

Baseline serum micronutrient data of overweight and obese individuals with a body mass index (BMI) between 25 and 40 kg/m^2^ and aged between 18 and 65 years was compared to the clinical micronutrient reference ranges for associations between BMI and micronutrient status.

**Results:**

There were significant negative associations between BMI and serum vitamin D (*p* = 0.044), folate (*p* = 0.025), magnesium (*p* = 0.010) and potassium (*p* = 0.023).

**Conclusions:**

Overweight and obesity appears to impact on the bioavailability and utilisation of micronutrients with absorption, excretion, storage/distribution (fat sequestering, tissue dispersion), metabolism (catabolic losses, possibly oxidative), increased physiologic requirements, and lower absolute total dietary intake being the current theory for observed differences. While vitamins D, folate, magnesium and potassium showed a negative relationship to BMI, other micronutrients did not. This may be explained by the fortification of certain processed foods, or the possibility of overweight and obese people eating more to satisfy their nutritional requirements.

## Background

In most cases, overweight or obesity is due to a positive energy balance stemming from excessive energy intake - compared to energy expenditure - from diets that are typically nutritionally poor [1]. Excess body weight has been shown to alter the absorption, distribution, metabolism, and/or excretion of micronutrients [2]. For example, vitamin D from cutaneous and dietary sources has been shown to have decreased bioavailability in obese persons and is potentially commandeered by adipose tissue [3]; thiamine metabolism is also impacted in obese persons, leading to a decrease in cellular absorption and an increase in intracellular conservation [4].

Micronutrient interactions within the food matrix can impact absorption and bioavailability by a number of mechanisms. Minerals with chemical similarities can compete for transport proteins or other uptake mechanisms, as well as for chelating organic substances, facilitating or hindering absorption [5]. The overall impact of these interactions will be determined by the relative concentrations of the nutrients. At normal dietary concentrations the absorption of most minerals is active or saturable, while at higher intakes passive diffusion can take place [5, 6]. A poor nutritional status with regard to vitamins affects mucosal integrity and can thereby affect absorption of other nutrients.

Nutrient reference values as provided by the National Health and Medical Research Centre (NHMRC) are ‘Recommended Dietary Intake’ (RDI) or Adequate Intake (AI), which includes the amounts of specific nutrients required on average each day for sustenance or avoidance of deficiency states [7]. Further nutritional advice is also provided in the form of national ‘Dietary Guidelines’. These guidelines provide culturally-relevant food and dietary patterns that will not only achieve nutritional balance, but also reduce the risk of nutrition related deficiency and chronic disease. When the diet of obese individuals is nutrient poor, the question arises as to whether micronutrient supply from consumed foods is sufficient to provide for biochemical and physiological demands. On the other hand, it may be that the high consumption of nutritionally poor foods may be driving the desire to consume more in order to attain nutritional sufficiency.

Few studies have examined the nutritional status of overweight and obese adults. One such study suggested vitamin D, chromium, biotin, thiamine and vitamin C levels are significantly lower in persons who are obese and that insufficiency in these micronutrients has the ability to impact glucose metabolism and cause insulin resistance [8]. Biochemical markers of nutritional status in overweight or obese populations have been previously studied, however these studies have often been limited by small sample sizes, or the examination of only a small number of micronutrients [9].

The current study examined baseline blood micronutrient levels of a group of overweight and obese Australian adults with the most up-to-date serum nutrient reference ranges for Australian adults. The aim of this study was to assess the nutritional status of overweight and obese Australian adults prior to the commencement of a high fibre weight loss clinical trial, to determine if there were any associations between BMI and poor serum micronutrient levels. Vitamins A, B_12_, C, D, E and B_9_ as well as minerals, iron, iodine, calcium, potassium, sodium, magnesium and zinc were evaluated.

### Research question

What is the baseline micronutrient status of a sample of overweight and obese Australian adults?

## Methods

### Participants

Overweight and obese individuals (*n* = 127) with a BMI between 25 and 40 kg/m^2^ and aged between 18 and 65 years, were recruited from the community in Perth, Australia via advertisements in newspapers, flyers posted around the University and community noticeboards, as well as radio advertising on Curtin FM. Potential participants were screened by telephone or online using a Qualtrics Survey and attended an orientation session at Curtin University to assess suitability for the study, at which time the details of the study were also explained. Exclusion criteria included smoking, lipid lowering medication, use of steroids and other agents that may influence lipid metabolism, diuretics, use of warfarin, diabetes mellitus, hypo and hyperthyroidism, cardiovascular events within the last 6 months, psychological unsuitability, major systemic diseases, gastrointestinal problems, proteinuria, liver, renal failure, weight fluctuations over the past 6 months, vegetarianism or veganism and participation in any other clinical trials within the last 6 months.

### Study design

The study design for the larger scale, long term dietary intervention study titled: Comparison of two different fibre supplements on body weight, body composition, metabolic and cardiovascular risk factors in overweight and obese individuals, is as follows: The study was randomised, controlled double blind, parallel design study over a 12-month period. Group allocation was randomised by the supplement supplier, who had no involvement in the trial, and double blinded to reduce bias or interference from the participants and the research assistants. Participants attended a briefing session on how to consume the supplements, complete the paperwork and comply with the study protocol. Dietary intake over the course of the trial was monitored through the completion of 3-day food diaries at each clinical visit (week 0, 12, 26 and 52). Participants in all groups were asked to maintain their usual diet for the duration of the study. For the purposes of this study only serum and food diary data obtained from week 0 was used for comparison.

## Assessments

### Micronutrients blood analysis

For the purposes of this present study, baseline blood samples were analysed for 127 participants. Volunteers attended Curtin University, after an overnight fast of 10–12 h, where blood samples were drawn by venepuncture. Samples were collected into lithium heparin or serum separator tubes (5 ml) for antioxidant/vitamins A, B_12_, B_9_, C, D and E and minerals (calcium, iron, iodine, magnesium, potassium, sodium and zinc) for analysis. All blood samples were collected by a trained phlebotomist. Blood samples were then centrifuged at 2500 rpm at 4 °C for 10 min using an Eppendorf centrifuge and prepared for storage at -80 °C. Micronutrients were analysed systematically, after all the participants had completed the 52-week study. Vitamins A, E, D, as well as vitamin B_9_, and B_12_ and thyroglobulin as a measure of Iodine status were measured using Enzyme-Linked Immunosorbent Assay kits (ELISA). ELISA combines antibody binding with enzymatic detection to identify molecules of interest; the result is a colour change that is measured by spectrophotometry at a particular wavelength [10]. Vitamin C was measured by colorimetric assay. All trace metals (calcium, magnesium, iron, zinc, sodium and potassium) were analysed by Flame Atomic Absorption Spectroscopy (FAAS). Atomic absorptiometry measures the concentration of gas-phase atoms or ions as a solution via the absorption of light after the solution is vaporized in a flame or graphite furnace [11].

### Outcome measures

The present study was designed to assess participants’ nutritional status in relation to: vitamins A, B_12_, B_9_, C, D E, folate and minerals calcium, iron, iodine, magnesium, potassium, sodium and zinc. Other outcome measures that formed part of the larger study, such as weight/BMI and metabolic syndrome risk factors, have been analysed and discussed elsewhere [12, 13].

### Statistical analysis

To determine the nutritional status of this sample (*n* = 127) of overweight/obese individuals, baseline micronutrients were observed with the clinical reference intervals (serum) sourced from the Stedman’s Medical Dictionary (established by the Harmonisation Committee of the Australasian Association of Clinical Biochemists (AACB)) or, where there was an absence of data, The Merck Manual [6, 14]. The reference range for thyroglobulin (as a marker of iodine status) however was taken from the assay manual as neither Stedman’s Medical Dictionary nor the Merck Manual had useful values for this micronutrient. The food diary micronutrient data were compared to the National Health and Medical Research Council (NHMRC) Nutrient Reference Values, which are the recommended dietary intake to maintain good health [15].

One sample t-tests were conducted, with the lower limit from the clinical reference range for each micronutrient used as the test value, with the exception of thyroglobulin (an indirect measurement of iodine status), where the upper limit was used [16]. The baseline data did not meet the assumption of normality for simple linear regressions (or one-sided Pearson’s bivariate correlation), so instead a one-side non-parametric bivariate correlation (eg Spearman’s) between BMI and each micronutrient was conducted. Statistical significance was considered at *p* < 0.05. All statistical analysis was conducted using SPSS 23.0 (IBM® SPSS® Statistics, New York, NY).

## Results

### Baseline characteristics

Blood samples, for the 127 overweight and obese participants, were analysed for baseline micronutrient levels. The group results showed that the average age of the study participant was 49.3 ± 1.0 years, the average weight was 94.0 ± 1.5 kg and average BMI was 32.3 ± 0.4 kg/m^2^. The gender of the study group was mostly female with 73 women versus 54 men. Of the 127 participants 33.1% were classified as overweight and 66.9% fell within the obese category according to BMI.

### Nutritional assessment

**Fig. 1:**
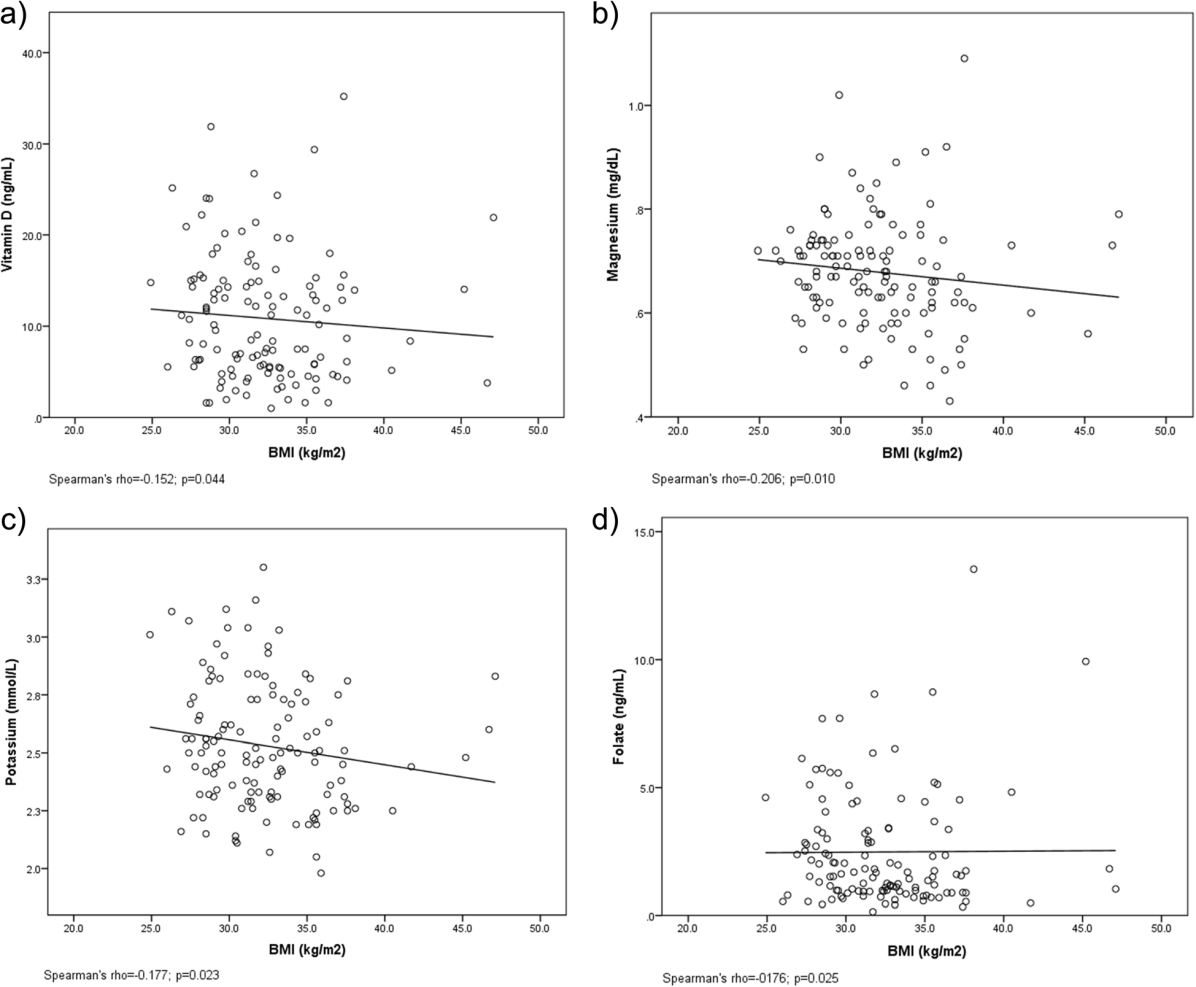
Significant associations between BMI and **a)** Vitamin D, **b)** Magnesium, **c)** Potassium, and **d)** Folate. All *p*-values are one-sided

Mean baseline serum and dietary intake data is outlined in Table 1. Dietary intake data was analysed using FoodWorks nutritional analysis software. For the purposes of this study iodine levels from blood samples were measured as serum thyroglobulin, as it is a more accurate biomarker for iodine deficiency [16]. Values were expressed as Mean ± SEM in their units of measurement for serum. Table 2 compares the Mean ± SEM serum values with the clinical reference intervals with values being either less than (<), within range or greater than (>) clinical reference intervals (14). Table 3 compares the dietary intake data analysed using FoodWorks nutritional software against the Nutrient Reference Values (NRV) for Australia and New Zealand [15]. Data from FoodWorks is not available for B_12_ and direct comparison between the NRVs and vitamin A, measured as retinol equivalents (RE) was not possible either as FoodWorks breaks vitamin A into retinol and beta carotene (refer to Table 4). Mean ± SEM serum values for micronutrients were also correlated with BMI (Table 4) and significant associations can be seen in Fig. 1a), b), c) and d). The relationship between self-recorded dietary intake and BMI was also examined as shown in Table 5, and there were no significant differences *p* > 0.05 found. As data analysis for vitamin B12 cannot be performed in FoodWorks, this micronutrient is not in Table 5.

**Table 1:** Baseline Characteristics of participants^a^

	Mean ± SEM	Median	*n*
Age (years)	49.3 ± 1.0	50	127
Weight (kg)	94.0 ± 1.5	89.9	127
BMI (kg/m^2^)	32.3 ± 0.4	31.8	127
Serum^a^			
Vitamin E (μg/mL)	7.79 ± 0.5	6.5	126
Vitamin B12 (pg/mL)	722.9 ± 41.3	707.8	126
Vitamin C (mg/dL)	3.7 ± 1.4	3.5	127
Vitamin A (μg/dL)	5.04 ± 0.2	5.6	127
Vitamin D (ng/mL)	10.9 ± 0.6	9.5	127
Folate (ng/mL)	2.5 ± 0.2	1.7	125
Thyroglobulin (ng/mL)	8.8 ± 1.3	5.9	119
Potassium (mmol/L)	2.5 ± 0.02	2.5	127
Sodium (mmol/L)	118.8 ± 0.9	117.6	127
Total Iron (μg/dL)	103.4 ± 3.3	100	127
Male	114.4 ± 4.8	110	54
Female	95.2 ± 4.4	90	73
Zinc (μg/dL)	27.9 ± 1.2	25	127
Calcium (mg/dL)	3.4 ± 0.1	3.3	127
Magnesium (mg/dL)	0.7 ± 0.01	0.7	127
Dietary Intake^b^			
Vitamin E (mg)	8.9 ± 0.4	7.9	127
Vitamin C (mg)	103.3 ± 6.9	76.9	127
Retinol (μg)	369.0 ± 23.8	322.6	127
Beta carotene (μg)	2681.5 ± 197.8	2134.2	127
Vitamin D (μg)	3.7 ± 0.2	3	127
Folate (μg)	373.2 ± 17.0	332.4	127
Iodine (μg)	128.4 ± 4.6	120.2	127
Potassium (mg)	3095.3 ± 73.1	3081.2	127
Sodium (mg)	2752.4 ± 100.0	2603.2	127
Iron (mg)	12.4 ± 0.4	11.6	127
Zinc (mg)	13.1 ± 0.5	12.3	127
Calcium (mg)	897.5 ± 34.6	869.1	127
Magnesium (mg)	373.4 ± 11.4	358	127

^a^Serum measurement units were converted to be consistent with the clinical reference values. ^b^Values derived from self-reported data

**Table 2:** Nutritional status of participants (serum)

Outcome	Reference	*n*	%
BMI (kg/m^2)^	> 25 overweight	42	33.1
	> 30 obese	85	66.9
Vitamin E (μg/mL)	< 5	39	31.0
	Within range	79	62.7
	> 18	8	6.3
Vitamin B12 (pg/mL)	< 110	2	1.6
	Within range	72	57.1
	> 800	52	41.3
Vitamin C (mg/dL)	< 0.4	0	0.0
	Within range	5	3.9
	> 1.5	122	96.1
Vitamin A (μg/dL)	< 30	127	100.0
	Within range	0	0.0
	> 80	0	0.0
Vitamin D (ng/mL)	< 20	113	89.0
	(No reference)	14	11.0
Folate (ng/mL)	< 3	90	72.0
	Within range	35	28.0
	> 20	0	0.0
Thyroglobulin (ng/mL)	< 2	16	13.4
	Within range	101	84.9
	> 50	2	1.7
Potassium (mmol/L)	< 3.5	127	100.0
	Within range	0	0.0
	> 5.1	0	0.0
Sodium (mmol/L)	< 136	120	94.5
	Within range	3	2.4
	> 145	4	3.1
Iron (μg/dL) - Male	< 65	3	5.6
	Within range	48	8.9
	> 175	3	5.6
Iron (μg/dL) - Female	< 50	4	5.5
	Within range	68	93.2
	> 170	1	1.4
Zinc (μg/dL)	< 70	126	99.2
	Within range	0	0.0
	> 120	1	0.0
Calcium (mg/dL)	< 4.64	116	91.3
	Within range	4	3.1
	> 5.28	7	5.5
Magnesium (mg/dL)	< 1.6	127	100.0
	Within range	0	0.0
	> 2.6	0	0.0

Measurement units were converted to be consistent with the clinical reference values. “<“may indicate deficiency, “>“may indicate excess, except for thyroglobulin, where a higher value may indicate iodine deficiency

**Table 3:** Self-recorded micronutrient status of participants at baseline compared to nutrient reference values

Outcome	Gender	Age (years)	Reference	Mean ± SEM	Median	n
Vitamin E (mg)	Male	19–70	10 (AI)	8.5 ± 0.6	8.1	54
	Female	19–70	7 (AI)	9.1 ± 0.6	7.9	72
Vitamin C (mg)	M/F	19–70	45 (RDI)	103.3 ± 6.9	76.9	126
Retinol (μg)^a^	Male	19–70	900 RE^a^ (RDI)	369.0 ± 23.8	322.6	126
Beta carotene (μg)^a^	Female	19–70	700 RE^a^ (RDI)	2681.5 ± 197.8	2134.2	126
Vitamin D (μg)	M/F	19–50	5 (AI)	3.6 ± 0.3	3.3	63
	M/F	51–70	10 (AI)	3.7 ± 0.7	2.8	63
Folate (μg)	M/F	19–70	400 (RDI)	373.2 ± 17.0	332.4	126
Iodine (μg)	M/F	19–70	150 (RDI)	128.4 ± 4.6	120.2	126
Potassium (mg)	Male	19–70	3800 (AI)	3156.3 ± 116.3	3207.4	63
	Female	19–70	2800 (AI)	3034.2 ± 88.8	3053.1	63
Sodium (mg)	M/F	19–70	460–920 (AI)	2752.4 ± 100.0	2603.2	126
Iron (mg)	Male	19–70	8 (RDI)	13.7 ± 0.7	13.0	54
	Female	19–50	18 (RDI)	11.4 ± 0.6	11.2	37
	Female	51–70	8 (RDI)	11.5 ± 0.7	10.6	35
Zinc (mg)	Male	19–70	14 (RDI)	14.3 ± 0.8	13.6	54
	Female	19–70	8 (RDI)	12.2 ± 0.6	11.2	72
Calcium (mg)	Male	19–70	1000 (RDI)	967.0 ± 64.6	896.7	54
	Female	19–50	1000 (RDI)	861.2 ± 52.9	869.4	37
	Female	51–70	1300 (RDI)	828.6 ± 48.0	797.6	35
Magnesium (mg)	Male	19–30	400 (RDI)	414.2 ± 46.0	444.8	3
	Male	31–70	420 (RDI)	395.4 ± 17.0	385.2	51
	Female	19–30	310 (RDI)	310 ± 58.5	301.6	6
	Female	31–70	320 (RDI)	360.2 ± 16.1	348.8	66

*AI* Adequate Intake, *RDI* Recommended Dietary Intake, *RE* Retinol Equivalents. Use of AI and RDI, as well as age and gender values, is based on currently available data. Incomplete data from FoodWorks nutritional analysis software means that direct comparisons between Retinol Equivalents and dietary intake couldn’t be made. ^a^1 μg Retinol Equivalent is equivalent to: 1 μg of all-trans retinol; 6 μg all-trans ß-carotene; or 12 μg of a-carotene, ß-cryptoxanthin and other provitamin A carotenoids [15]

**Table 4:** Correlation between BMI and serum micronutrients of participants in the study

	*n*	Correlation Coefficient	Sig. (1-tailed)
Vitamin E	126	−0.019	0.418
Vitamin B12	126	−0.093	0.150
Vitamin C	127	−0.133	0.068
Vitamin A	127	0.078	0.190
Vitamin D	127	−0.152*	0.044*
Folate	125	−0.176*	0.025*
Iodine	119	−0.067	0.236
Potassium	127	− 0.177*	0.023*
Sodium	127	0.092	0.151
Iron	127	−0.122	0.086
Zinc	127	−0.034	0.352
Calcium	127	−0.087	0.166
Magnesium	127	−0.206*	0.010*

*Indicates significance at *p* < 0.05

**Table 5:** Relationship between dietary intake and BMI

	*n*	Correlation Coefficient	Sig. (1-tailed)
Vitamin E	126	0.018	0.419
Vitamin B12	N/A	N/A	N/A
Vitamin C	126	−0.049	0.294
Retinol	126	−0.110	0.111
Beta Carotene	126	0.024	0.396
Vitamin D	126	0.038	0.335
Folate	126	−0.005	0.478
Iodine	126	0.119	0.092
Potassium	126	0.035	0.35
Sodium	126	−0.118	0.093
Iron	126	0.048	0.297
Zinc	126	−0.023	0.400
Calcium	126	0.014	0.436
Magnesium	126	0.096	0.142

*There were no significant between group differences *p* < 0.05. Data analysis for vitamin B12 cannot be performed in FoodWorks

### Correlations

Baseline serum and dietary micronutrient concentrations of the participants, are shown in Table 1). Mean and median values of this population are shown, however for the purposes of statistical analysis, median values were used. The data was not normally distributed due to outliers so median values were seen as the most accurate representation and more likely to be applicable to the wider community than mean values.

The baseline serum values were compared with the clinical reference intervals for nutrients in Australia (AACB) [17]– shown in Table 2). Serum values were either ‘lower than the recommended range (<)’ indicating a deficit of that particular nutrient, ‘within range’ indicating sufficiency or ‘greater than the recommended range (>)’ indicating excess. The results (Table 2) showed that 62.7% of the participants in the study were within the healthy reference range for dietary vitamin E (5–20 μg/mL), with a mean value of 7.79 ± 0.5 μg/mL. For vitamin B_12_ 57.1% of the sample population in the study were within range where as 41.3% were in excess of the reference range with mean levels at 722.9 ± 41.3 pg/mL. Vitamin C showed that 96.1% of the sample were in excess of the reference range whereas 100% of subjects did not meet the clinical reference interval for vitamin A; results were considerably less that the reference range of 28–86 μg/dL with the mean sample value only 5.04 ± 0.2 μg/dL. The majority (89%) did not reach required levels for vitamin D, deficiency is described at <20–24 ng/mL and the sample mean was 10.9 ± 0.6 ng/mL. Only 28% were within the reference range for folate, with deficiency <3 μg/L, the sample mean was 2.5 ± 0.2 μg/L. However, thyroglobulin levels were mostly (84.9%) within range 2–50 ng/ml with a mean of 8.8 ± 1.3 ng/mL. 100% of the sample did not meet the NRV for potassium with deficiency at <3.5 mEq/L; and the sample mean: 2.5 ± 0.02 mmol/L. Sample population serum values for sodium (Mean 118.8 ± 0.9 mmol/L), zinc (Mean 27.9 ± 1.2 μg/dL) and calcium (3.4 ± 0.1 mg/dL) levels were all lower than the clinical reference interval. The majority of participants were within the clinical reference interval for iron with a reference of 30 to 300 ng/mL for serum ferritin and mean values for men 114.4 ± 4.8 μg/dL and women 95.2 ± 4.4 μg/dL respectively. Serum magnesium levels were significantly lower than the clinical reference interval with a mean value of 0.7 ± 0.01 mg/dL compared to 1.8–2.6 mg/dL.

Table 3) shows micronutrient status of participants at baseline using self-reported dietary intake data compared to NRVs for Australia and New Zealand (NHMRC). Table 3) indicates that recommended nutritional intake from the diet is being met for some micronutrients, but not for others. Vitamin E is measured as adequate intake (AI) per day for 19–70 years old’s, and for men the reference is 10 mg while for women its 7 mg per day. The study participants were under the reference value for men (8.1 mg) and slightly over the reference value for women (7.9 mg). The RDI for vitamin C is 45 mg daily for 19–70 year old’s; the study participants well exceeded this RDI at 76.9 mg. For vitamin A, retinol equivalents (RE) are the reference used. Retinol (μg) RDI is 900 RE and 700 RE for beta carotene (μg). Study participants were under the recommendations for retinol with a median value of 322.6 μg and over the RDI for beta carotene at 2134.2 μg. For vitamin D, both men and women were under the AI for both age groups, with dietary intake data showing only 3.3 μg for 19–50 years compared to the recommended 5 μg required daily and 2.8 μg for 51–70 years old compared to 10 μg required. The mean dietary folate intake was just under the 400 μg recommended intake value at 332.4 μg; similarly the mean iodine intake was 120.2 μg, which is below the RDI of 150 μg. For potassium, males were below the AI of 3800 mg daily with a median value of 3207.4 mg, whereas females exceeded AI of 2800 mg with 3053.1 mg. Dietary intake for sodium well surpassed the AI of 460–920 mg daily with 2603.2 mg recorded. Males met the RDI for iron of 8 mg per day. Females of 19–50 years of age were under the recommended 18 mg daily with 11.2 mg, but exceeded the RDI for females aged 51–70 years. The zinc RDI for 19–70 years old was met for females but slightly under recommendations for males at 13.6 mg instead of 14 mg. Both males and females did not meet the RDI for calcium, however females were further below the RDI than males. For the 19–50 year age group, data for females showed a daily intake of 869.4 mg/day compared with the 1000 mg recommended. The 51–70 year age group showed a greater deficit with 797.6 mg compared to 1300 mg RDI. The magnesium RDI was met for males in the 19–30 year age category but below the RDI for females at 301.6 mg instead of 310 mg. However the reverse was true for the 31–70 year age category, with females exceeding the RDI and males only achieving a median value of 385.2 mg instead of the recommended 420 mg.

For Table 4) Correlation between BMI and serum micronutrients for participants at baseline, significant associations (Spearman’s rho) were found for vitamin D (r_s_ = − 0.152, *p* = 0.044), folate (r_s_ = − 0.176, *p* = 0.025), potassium (r_s_ = − 0.177, *p* = 0.023), and magnesium (r_s_ = − 0.206, *p* = 0.010). Vitamin D, folate, magnesium and potassium all showed that as BMI increased the serum concentration for these micronutrients decreased slightly (refer to Fig. 1) a, b c) and d). The line of best fit in Fig. 1a), b) and c) all show a downward trend which correlates with the negative r-values obtained for these micronutrients. However, the line of best fit for folate appears to show a slightly positive association with increased BMI, even though the r-value is negative (Fig. 1d). The line of best fit should show a downward trend as BMI increased similar to the other micronutrients, but the slight upward trend appearance may be due to possible BMI outliers skewing the trend line making it appear slightly positive. Correlations for all other serum micronutrients with BMI were not considered statistically significant.

For Table 5) Relationship between self-recorded dietary intake data and BMI showed no significant associations at *p* < 0.05.

## Discussion

Overweight and obesity is one of the predominant health issues in todays’ global society, having superseded malnutrition in recent years [18, 19]. The diet of an overweight or obese individual is typically energy dense and nutrient poor, thus low micronutrient levels may result from inadequate dietary intake and/or alterations in nutrient absorption or metabolism over time [18]. Baseline serum micronutrient results for study participants indicated that vitamin D, magnesium, potassium and folate status appeared to be affected by BMI. When serum and dietary intake data was compared to the clinical reference intervals and the Nutrient Reference Values for Australia and New Zealand, many micronutrients were also outside normal ranges or recommendations.

This study found a negative correlation (Spearman’s rho r_s_ = − 0.152, *p* = 0.044) between serum vitamin D status and body mass index (refer to Table 4 and Fig. 1a), with the majority (89%) not reaching the required levels for Vitamin D which is 5 μg/day for 19–50 year old Australian adults (refer to Table 3). Vitamin D deficiency is noted in serum levels less than 50 nmol/L, with clinical reference ranges from 60 to 160 nmol/L [20]. Most vitamin D is obtained from sun exposure, however dietary intake of vitamin D containing foods is still important. Oily fish such as salmon and mackerel, eggs, mushrooms and fortified foods are among the highest vitamin D containing foods [7, 20]. Baseline dietary intake data from study participants was compared to the NRV’s for Australia and New Zealand (Refer to Table 3) and it was found that vitamin D obtained from dietary sources was below the daily recommended amount. For the 19–50 year age group 5 μg is the AI per day, while the mean showed only 3.6 ± 0.3 μg was obtained from dietary sources. For 51–70 year old’s the AI for vitamin D increases to 10 μg per day, however self-recorded intake showed a significant deficit at only 3.7 ± 0.7 μg per day. This could help explain why serum vitamin D levels were below the clinical reference intervals (refer to Table 2) with 89% of participants having serum levels less than 20 ng/mL.

Vitamin D has also been shown to have decreased bioavailability from cutaneous and dietary sources in overweight and obese populations, as it is potentially sequestered by adipose tissue [21]. This may explain significant negative association between BMI and serum vitamin D levels during this study. A study by Sadiya et al. demonstrated that large amounts of vitamin D3 is stored in adipose tissue after vitamin D3 supplementation, and suggests that overweight and obese participants may store more vitamin D than healthy weight participants because they have larger amounts of adipose tissue [22]. An Australian study by Gill, et al. 2014 found a direct correlation between BMI and vitamin D status, indicating that those with a BMI >25 had lower serum vitamin D than those with a BMI < 25. Secondly, those who undertook regular physical activity had higher serum vitamin D than those who were inactive [20]. Seasonal changes in vitamin D levels are also prevalent with lower serum vitamin D3 levels (≤ 50 nmol/L) found in winter and spring [23]. Due to the large sample size and the rolling roster of clinical appointments, some participants baseline values were obtained during summer, autumn and winter, which may have resulted in some variability in the findings [23]. While seasonal variations are important, it could be argued behavioural changes are more significant. In summer, although participants are more inclined to have more skin exposed, they are also more likely to wear sunscreen and a hat due to the harsh Australian sun and the associated increase in skin cancer risk [24]. A large study by Daly et al. examined serum vitamin D in 11,247 samples collected Australia wide; results showed that vitamin D deficiency (< 50 nmol/L) to be common in adults aged 25 years and over (31% of total sample), with women, those with obesity, the elderly, those from a non-European background and those with insufficient physical activity levels being most at risk of low serum vitamin D [24].

Table 4) shows that serum magnesium levels were also lower in those with higher BMIs (see Fig. 1b). This finding is further supported by all participants being outside normal serum parameters of the clinical reference intervals (Refer to Table 2). Typically green leafy vegetables, nuts, seeds, legumes and whole grains, are good sources of magnesium [7]. The RDI for Australians 19–30 years of age is 400 mg for men and 310 mg per day for women, and for those 31–70 years of age its 420 mg for men and 320 mg for women (Table 3). When using the self-reported dietary intake data and comparing the mean values to the NRVs, dietary intake of magnesium was being met. However this did not translate to serum magnesium with all participants being below the clinical reference intervals (Table 2). It is estimated only 30 to 40% of dietary magnesium is absorbed by the body, so regular intake is essential [25]. Previous studies have linked obesity, and obesity-related metabolic risk factors such as glucose intolerance, cardiovascular disease, dyslipidaemia and insulin resistance, with low serum magnesium [26]. Low dietary intake of magnesium, impairs intestinal absorption and promotes higher circulating inflammatory markers in overweight and obese individuals [27]. Intestinal inflammation is known to impair micronutrient absorption [28]. There is also a correlation between lower serum magnesium and low vitamin D levels in overweight and obese individuals. Although a magnesium-regulating hormone or factor has yet to be described, the effect of vitamin D on serum magnesium concentration has been confirmed in some studies [29]. A study by Farhanghi, et al. 2009 showed that low baseline concentrations of serum magnesium in obese participants can induce higher renal magnesium retention following vitamin D supplementation [27]. The injection of the metabolite of 1,25 (OH)2 vitamin D (equivalent to 600,000 IU) given in this study acted as a strong modifier of magnesium in participants with baseline serum concentrations lower than 1 meq/l. While this may explain lower magnesium and vitamin D concentrations in those with higher BMI in the current study, measuring total body magnesium status accurately can be a challenge as serum magnesium represents approximately 1% of total body Mg, which may reflect renal handling rather than dietary intake [25].

Significantly lower baseline potassium concentrations in participants with a higher BMI was also found (Table 4 and Fig. 1c). Potassium is an intracellular cationic electrolyte, necessary for normal cellular function [30]. Potassium is not stored in the human body, but excreted by the kidneys, therefore a regular dietary supply is required [31]. Several studies have suggested a correlation between potassium and central adiposity, which is a risk factor of metabolic syndrome (MS) [31], however the precise relationship is unclear. Current evidence suggests that overweight and obesity alters potassium channel function [30]. Potassium plays a critical role in insulin secretion, hypertension and carbohydrate metabolism, and can affect carbohydrate accumulation and glucose homeostasis [31]. However, adequate potassium intake appears to have a protective effect on obesity. Fruit and vegetables are a major source of dietary potassium, thus a high intake would also be beneficial to MS risk factors such as central adiposity [30]. Dietary intake data in Table 3 showed that males were slightly below the NRV for potassium at 3800 mg per day, whereas females exceeded the NRV of 2800 mg per day. Potassium is very well absorbed by the body with about 90% absorbed from dietary sources [15]. However the serum potassium levels for all study participants as compared to the clinical reference intervals (Table 2) was below the acceptable range of 3.5–5.1 mmol/L. Assessing potassium levels via serum is not the best indication of potassium status because most potassium in the body is stored inside cells. Although serum levels can provide some indication of potassium status, they are a poor reflection of tissue potassium stores [7, 30].

Baseline serum folate levels also correlated negatively with BMI (Refer to Table 4 and Fig. 1d). The form of folate used in supplements and food fortification is folic acid. Folate and folic acid is found in dark green leafy vegetables, legumes, fortified cereals and foods and has a bioavailability of 50–85% depending on the food and form consumed [32]. Serum folate levels as shown in Table 2), indicated that 72% of participants were below the clinical reference interval and 28% were within normal reference range for serum. Dietary intake data (Table 3) showed that males and females were just under the RDI for folate of 400 μg per day at 373.2 ± 17.0 μg. Recent studies on folic acid fortification have revealed that individuals with obesity present low fasting serum but high erythrocyte folate concentrations, as well as high levels of serum folate oxidation products [33]. It has been shown that high erythrocyte folate status can reflect long-term excess folic acid intake; increased folate oxidation products are correlated with increased folate degradation as obesity can result in increased cytochrome P450 2E1 activity. Cytochrome P450 2E1 is a monooxygenase enzyme that can use folic acid as a substrate [33]. This clarifies why folate status decreases as BMI increased, and why folate status is impacted negatively by being overweight or obese.

Calcium intake of the majority of participants was also below the reference value recommendation (Table 3). In addition, serum calcium levels were well below the clinical reference interval for 91.3% of participants (See Table 2), however within this study there was no correlation between calcium and BMI. Calcium intake recommendations from food sources such as dairy products, nuts, green leafy vegetables, and fortified foods and milks, for Australian adults is 1000 mg/day for adults between 19 and 50 years of age and 1300 mg/day for older groups [15]. According to the Australian Health Survey 2011–2012, over half of the Australian population aged 2 years and over had inadequate usual intakes of calcium [34]. Low calcium intake is considered a risk factor for certain disorders, including osteoporosis, hypertension, cancer, insulin resistance, and the metabolic syndrome [35]. Interestingly, low dietary calcium intake was listed among the risk factors significantly associated with overweight and obesity in a number of published studies [36, 37] and there appears to be a direct link between high dietary calcium levels and increased faecal fat excretion. The proposed mechanism by which calcium may contribute to a negative energy balance is the formation of insoluble calcium/fatty-acid soaps, which pass unabsorbed through the intestinal tract and are excreted in the faeces [38, 39], appetite control as demonstrated from intervention studies involving dairy calcium supplementation [39] and cellular mechanisms such as the mobilisation and oxidation of fats [40]. Low dietary calcium can lead to an elevated cytosolic calcium and free ionic calcium in the cytosol plays a significant part in metabolic disorders related to insulin resistance and obesity [40].

Serum sodium levels were also significantly lower than the recommended clinical reference interval (Table 2), however exceeded the NRVs in the 3-day dietary intake data (Table 3). Sodium levels within the body are maintained within a narrow range of 135 to 145 mEq/L, and the mechanisms which maintain the plasma sodium concentration in a narrow range are thirst and antidiuretic hormone (arginine vasopressin) release [41]. Hyponatremia (low sodium) indicates hypotonicity - water excess for the amount of sodium present [41]. Participants were instructed to fast prior to clinical visits, however were still allowed to drink water to maintain hydration for ease of venepuncture. No fasting obviously occurred during recording of the 3-day food diaries completed by participants, so this may explain why self-recorded dietary intake versus serum sodium was so different. The low serum sodium levels obtained from study participants at baseline may be due to simple dilution of serum prior to blood samples being taken, especially as the dietary intake data shows that participants were exceeding the NRV for sodium, however this dilution theory is not supported by the available literature.

Micronutrients (and macronutrients) have been implicated as an important factor in regulating various metabolic processes and thus playing a role in the aetiology of obesity. Many studies are being conducted worldwide that clearly show a direct link between obesity and micronutrient deficiencies [21, 42]. Deficiencies of various micronutrients, such as fat-soluble vitamins, B complex, vitamin C and ions such as calcium and magnesium, have been associated with increased BMI as overweight or obese is synonymous with an energy dense, nutrient poor diet [8]. Current research shows that micronutrients play a crucial role in bioavailability and absorption of nutrients in the gastrointestinal tract, as well as regulate the hunger/satiety hormones [8]. If dietary insufficiency is ongoing then deficiency states may present, however this is potentially offset by food fortification of vitamins and minerals in a variety of processed foods and beverages.

In an ideal world, micronutrient requirements would be met by a varied diet high in fruit and vegetables. However several of studies have shown that people simply are not consuming the amounts required to achieve and maintain nutritional sufficiency [34]. In many cases, micronutrient food fortification, which involves adding specific nutrients to flour, cereals, processed or ready to eat foods, infant formula fortification, and vitamin-enriched drinks, has been used to treat and prevent nutritional deficiency diseases in populations at risk [43]. Many industrialized countries have used fortification to prevent deficiencies of vitamins A and D, several B vitamins (thiamine, riboflavin and niacin), iodine and iron [43]. In addition, vitamins and minerals may be obtained easily from artificial sources, such as nutritional supplements [44].

Key findings from the 2011–2012 Australian health Survey support that notion. Usual Nutrient Intake data showed that 73% of females and 50% of males aged 2 years and over did not meet their calcium requirements; 17% of males and 14% of females had inadequate usual intakes of vitamin A. One in eleven adult females (aged 19 and over) did not meet the requirement for folate, however almost all males let the dietary requirement through intake; insufficient B_12_ intake accounted for between 5 and 8% of females dependent on age and less than 1 % of males; 40% of 14–18 year old females and 38% of 19–50 year-old females had inadequate iron intakes compared to only 3% of males; less than 5% of the population did not meet their dietary needs for vitamin C and vitamin E; and, from age 14 males have a much higher requirement of zinc than females due to its key role in the male reproductive system [45]. According to the Australian Health Survey results 37% of men and one in ten women (9%) had inadequate usual zinc intakes [46]. One in every three people aged 2 years and over (37% of males and 34% of females) did not meet their requirements for magnesium, however 76% of males and 42% of females aged 2 years and over exceeded the UL for sodium [46].

When comparing the current study with the Australian Health survey findings (Table 2), 31% of participants had lower than required vitamin E levels, whereas 41% exceeded the requirement for B_12_. All of the study participants either met or exceeded vitamin C levels and for vitamin A every person had less than adequate serum levels. Eighty nine percent of participants had vitamin D levels that would put them in the ‘severe’ deficiency category. Seventy two percent were below the lower limit for folate, however 84.9% were within range for thyroglobulin which is a more accurate biomarker of folate status. For potassium, all participants measured had inadequate serum levels and 94.5% had lower than normal sodium levels. Approximately 90% (or 88.9% of males and 93.2% of females) were within reference range values for iron, however 99.2% had less than adequate serum values for zinc. Calcium values of the participants were also lower than the clinical reference values with 91.3% having a serum calcium level lower than < 4.64 mg/dL.

### Implications of associations

The exact mechanisms that explain the relationship between folate metabolism and obesity are still being established. Previous studies have reported people with higher BMI have high erythrocyte folate concentrations, as well as high levels of circulating serum folate oxidation products, but can also have low fasting serum levels [47]. Overweight and obesity are associated with epigenetic changes such as abnormal DNA methylation patterns for genes involved in metabolic regulation [47]. Significant associations between serum folate, DNA methylation, BMI, and body fat percentage have been reported [48]. This supports the results obtained in this study as a negative correlation between BMI and baseline fasted serum folate levels was found. The current hypothesis is that higher folate intake could act as a protective factor against obesity by epigenetic mechanisms, thus a poor folate status could contribute adversely to an individual’s weight [47]. A prolonged deficiency in folate can lead to folate deficiency anaemia (megaloblastic) and pancytopenia. Glossitis, angular stomatitis and oral ulcers as well as neuropsychiatric manifestations such as depression, insomnia, and psychosis are also known to occur with folic acid deficiency [32]. Neural tube defects are also a concern for the foetus of a pregnant woman with an insufficient folate intake [49].

A negative association with BMI for vitamin D, potassium and magnesium, implies people with higher BMI are at risk of deficiency and deficiency related conditions. Vitamin D, potassium and magnesium all play essential roles in bone health and calcium storage, resorption and regulation [22, 50]. A study by Sadiya et al. demonstrated that large amounts of vitamin D3 is stored in adipose tissue and suggests that overweight and obese participants may store more vitamin D than healthy weight participants because they have larger amounts of adipose tissue [22]. There is also a correlation between lower serum magnesium and low vitamin D levels in overweight and obese individuals. Vitamin D plays a role in renal magnesium retention, thus low Vitamin D would contribute to low magnesium levels [25, 27]. Current evidence suggests that overweight and obesity alters potassium channel function, however this mechanism is not currently well understood [51]. However a relationship between low potassium and central adiposity has been established [31]. Assessing potassium and magnesium levels via serum is not the most accurate measure of micronutrient status as most potassium in the body is stored inside cells and serum magnesium represents approximately 1% of total body Mg, however serum magnesium and potassium results do still provide reliable information on overall micronutrient status. Prolonged deficiencies of these micronutrients can lead to osteoporosis and poor bone health for vitamin D [24]. For magnesium and potassium deficiency symptoms are similar and include, fatigue, numbness, tingling, cramps, seizures, personality changes, abnormal heart rhythms, and coronary spasms can occur [52, 53]. Severe deficiency can also result in hypocalcemia because mineral homeostasis is disrupted [54].

### Strengths and limitations

This study is unique in the fact that not many studies have examined the nutritional status of overweight and obese individuals, as most focus on over-nutrition in terms of macronutrients, and interventions to treat this disease. The baseline figures suggest show that serum micronutrient levels for vitamin D, calcium, folate, potassium, sodium, magnesium and vitamin A were all below the NRV recommendation. The study participants who fall below the clinical reference intervals for these micronutrients could be said to have high calorie malnutrition. There is an emerging theory that proposes overweight and obese individuals who consume a large amount of highly processed food within the standard ‘Western Diet’ have a physiological drive to eat excessive amounts due to the poor nutritional content of these foods, and the body’s innate need to achieve (micro) nutritional sufficiency [55]. This study provides some useful data on another aspect of the overall health of this population subgroup.

Possible limitations of the current study may include that the blood samples and dietary intake data from 3-day food diaries were only collected at baseline whereas multiple time periods throughout the year may have been of more significance. Many deficiency states happen over a period of time depending on the micronutrient, so analysis of a ‘snap shot’ on the time scale may not be the most representative of micronutrient levels. Some also argue serum measurements aren’t the most accurate assessment of nutritional status for every micronutrient. Calcium status for example, bone mineral density is potentially a more accurate measure than serum calcium levels as serum calcium is so tightly regulated within the blood. Consequently if serum calcium levels fluctuate this is not necessarily an indication of status, but rather something is affecting bone calcium release or renal processing and reabsorption [56]. Also in this study, iodine status is measured by assessing thyroglobulin levels in the blood. Higher thyroglobulin levels suggest that the thyroid is working harder to compensate for low iodine levels and may be an indication of iodine deficiency [16]. However some experts suggest urinary iodine tests are a more accurate measure of status. For the purposes of the confines of this study, serum measurement of micronutrients was the best method available at the time. Misreporting by participants also had the potential to introduce confounders for this study. Including under reporting or not enough detail recorded by participants of food diaries, consumption of multivitamins that weren’t recorded, starting medications that may interfered with the data.

### Significance

With studies of overweight and obese individuals, it is not often that their baseline nutritional status is examined. By comparing serum micronutrient levels against the clinical reference intervals for Australia, it shows that dietary intake affects nutritional status and not just body weight, further highlighting the importance of following dietary recommendations for fruit and vegetables.

## Conclusion

There is limited research examining micronutrient status in individuals with overweight and obesity, and it is probable that different mechanisms explain nutrient-specific increases or decreases among the overweight and obese. Several hypotheses have been proposed to explain the variability of serum micronutrient levels in this subset of the population, including: how a vitamin or mineral is absorbed, excreted, stored/distributed; whether its sequestered by fat or dispersed in tissue, metabolic processes (catabolic loses, possibly oxidative), increased physiologic requirements, and lower absolute total dietary intake [4, 9]. Overeating to compensate for a nutritionally poor diet and food fortification may also explain why serum micronutrient levels are not more significantly affected in overweight and obese Australian adults. In addition, the interrelationship between overweight and obesity and increased risk of associated comorbidities such as cardiovascular disease, atherosclerosis, hypertension and myocardial infarction versus the potential decreased risk associated with adequate intakes of certain micronutrients especially magnesium and potassium [30, 57].

This study aimed to identify potential relationships between BMI and baseline micronutrient status, and found that participants within this sample with a higher BMI tended to have lower serum vitamin D, folate, magnesium and potassium levels. Serum micronutrient levels compared to the clinical reference intervals and dietary intake data compared to the NRVs also supported the theory that diet quality in this subset of the population is poor. More research is needed to further clarify the associated health implications of micronutrient deficiency or surplus in overweight and obese individuals.

## Data Availability

The datasets used and/or analysed during the current study are available from the corresponding author on reasonable request.

## Abbreviations

AACB: Australasian Association of Clinical Biochemists
AI: Adequate intake
ANCOVA: One way analysis of covariance
BMI: Body mass index
BP: Blood pressure
CHD: Coronary heart disease
CNS: Central nervous system
CVD: Cardiovascular disease
DF: Dietary fibre
DNA: Deoxyribose nucleic acid
ELISA: Enzyme-linked immunoabsorbant assay
FAAS: Flame atomic absorption spectroscopy
HDL-C: High density lipoprotein cholesterol
LDL-C: Low density lipoprotein cholesterol
MS: Metabolic syndrome
NHMRC: National Health and Medical Research Council
NIDDM: Non-insulin dependent diabetes mellitis
NRVs: Nutrient reference values
NSP: Non starch polysaccharide
PGX: PolyGlycopleX®
RBC: Red blood cell
RDIs: Recommended dietary intake
RE: Retinol equivalents
SCFA: short-chain fatty acid
SEM: Standard error of mean
TBW: Total body water
UL: Upper level
